# Tricuspid Regurgitation: Pathophysiology, Risk Stratification, and Implications for Intervention

**DOI:** 10.3390/jcm15103622

**Published:** 2026-05-08

**Authors:** Mariagrazia Piscione, Barbara Pala, Dario Gaudio, Paola Gualtieri, Mario Laudazi, Simone Steffani, Marcello Chiocchi, Ferdinando Iellamo, Francesco Giuseppe Garaci, Marco Alfonso Perrone, Laura Di Renzo

**Affiliations:** 1 Asl 2 Abruzzo, Santissima Annunziata Hospital, 66100 Chieti, Italy; mariagraziapiscione@gmail.com; 2 Renzetti Hospital, 66034 Lanciano, Italy; 3 PhD School of Applied Medical-Surgical Sciences, Tor Vergata University of Rome, Via Montpellier 1, 00133 Rome, Italy; 4 UOC Cardiologia Ospedale IDI—IRCCS, 00167 Rome, Italy; 5 Fondazione Policlinico Campus Bio-Medico, University of Rome, Via Alvaro del Portillo 200, 00128 Rome, Italy; dario.gaudio@unicampus.it; 6 Section of Food Science, Clinical Nutrition and Pharmaceutical Sciences, Department of Biomedicine and Prevention, Tor Vergata University of Rome, Via Montpellier 1, 00133 Rome, Italy; paola.gualtieri@uniroma2.it (P.G.); laura.di.renzo@uniroma2.it (L.D.R.); 7 Department of Biomedicine and Prevention, Diagnostic Imaging Division, Tor Vergata University of Rome, Via Cracovia 50, 00133 Rome, Italy; mariolauda@gmail.com (M.L.); simone.soldato94@gmail.com (S.S.); marcello.chiocchi@gmail.com (M.C.); francesco.garaci@uniroma2.it (F.G.G.); 8 Division of Cardiology and CardioLab, Department of Clinical Sciences and Translational Medicine, Tor Vergata University of Rome, Via Montpellier 1, 00133 Rome, Italy; iellamo@uniroma2.it (F.I.); marco.perrone@uniroma2.it (M.A.P.)

**Keywords:** tricuspid regurgitation, right heart failure, risk score, multimodality imaging, TRISCORE

## Abstract

**Background:** Right heart failure (HF) and tricuspid regurgitation (TR) are closely interrelated conditions, linked by a bidirectional and self-perpetuating pathophysiological relationship. Alterations in right-ventricular (RV) loading conditions, pulmonary vascular impedance, and ventriculo-arterial (VA) coupling play a central role in the development and progression of TR, which in turn exacerbates RV volume overload and end-organ dysfunction. **Methods:** This review provides a comprehensive overview of the pathophysiology of right HF and TR, focusing on the mechanisms underlying RV dysfunction, pressure–volume (PV) relationships, and pulmonary vascular load. We further examine the clinical implications of this interaction and summarize current strategies for risk stratification, with particular emphasis on disease-specific risk models. **Results:** TR emerges both as a consequence and a driver of RHF. Conditions such as pulmonary hypertension (PH) and left-sided heart disease promote annular dilation and leaflet tethering, leading to functional TR. Conversely, TR increases RV volume overload, worsening chamber dilation, reducing effective forward stroke volume (SV), and accelerating disease progression. This vicious cycle results in progressive RV impairment, impaired left-ventricular filling through ventricular interdependence, and systemic venous congestion affecting renal and hepatic function. Traditional risk scores fail to capture this complex pathophysiology. In this context, TRISCORE integrates clinical, biological, and echocardiographic (TTE) parameters reflecting RV dysfunction and systemic involvement, providing a more comprehensive assessment of disease severity and prognosis. **Conclusions:** TR should be considered not only a marker but also a key determinant of right HF progression. A multiparametric approach integrating pathophysiology and disease-specific risk stratification is essential to identifying the optimal therapeutic window and guiding clinical decision making.

## 1. Introduction

The tricuspid valve (TV) is a complex structure composed of the annulus, leaflets, and subvalvular apparatus. It is closely related to the right ventricle (RV), right atrium, and conduction system, which is relevant during surgical and transcatheter interventions (TTVI) [1,2].

Its dynamic three-dimensional geometry is highly sensitive to changes in RV loading conditions, including annular dilation and leaflet tethering, which are central to the development of functional tricuspid regurgitation (TR) [3,4,5,6].

For many years, isolated TR has been regarded as the “forgotten valve” disease, despite growing evidence linking significant TR to increased morbidity and mortality [7,8,9].

Over the last decade, epidemiological studies have demonstrated that moderate-to-severe TR is independently associated with reduced survival and a higher risk of heart failure (HF) hospitalization [10]. Nevertheless, surgical intervention for isolated TR remains relatively uncommon and is often performed at advanced stages of disease [11].

Importantly, isolated tricuspid valve surgery (ITVS) has historically been associated with substantial perioperative risk, with in-hospital mortality rates ranging between 8% and 10% in large surgical registries [11,12]. However, recent data suggest that these unfavorable outcomes may be driven not only by procedural complexity but also by late referral and advanced systemic involvement at the time of intervention [12].

Accordingly, one of the major unresolved issues in the management of TR is the identification of the optimal timing for intervention. Early intervention may expose patients with mild symptoms and preserved RV function to procedural risks without clear survival benefit [13,14,15]. Conversely, delaying intervention until advanced right HF develops may result in correction of valvular incompetence without meaningful reversal of systemic congestion and organ dysfunction [15]. These considerations highlight the need to identify the optimal “therapeutic window” in which intervention is most likely to confer clinical benefit.

In parallel, the rapid development of contemporary TTVI has expanded the therapeutic landscape, making patient selection increasingly relevant and reinforcing the need for clinically meaningful risk stratification tools.

The aim of this review is, first, to frame TV disease within the broader context of right HF and subsequently to provide an overview of the currently validated risk scores that may assist in guiding therapeutic decision making.

In this context, TR should not be considered solely a valvular disorder but rather a complex syndrome, in which procedural candidacy and outcomes are not determined solely by the degree of regurgitation.

Rather, a broad spectrum of anatomical, functional, and clinical factors—including RV function, right-atrial remodeling and pulmonary hemodynamics—critically influence both the feasibility of intervention and its likelihood of success.

## 2. Narrative Review Methodology

This narrative review was conducted to provide a clinically oriented overview of tricuspid regurgitation (TR), with a focus on pathophysiology, risk stratification, and contemporary management strategies. The relevant literature was identified through consultation of major biomedical databases, including PubMed/MEDLINE, using combinations of keywords such as “tricuspid regurgitation”, “right ventricular dysfunction”, “risk stratification”, “TRISCORE”, “transcatheter tricuspid valve intervention”, and “right heart failure”. Additional relevant studies were identified through manual review of reference lists from selected articles and key publications in the field. The literature was considered based on its clinical relevance and its contribution to the understanding of the topic, with particular emphasis on landmark studies and recent evidence. This approach was intended to provide a representative and clinically meaningful synthesis rather than an exhaustive or systematic assessment of all the available literature. Accordingly, this work should be considered a narrative review, and no formal systematic review methodology or structured risk-of-bias assessment was applied.

## 3. Pathophysiology of Right Heart Failure and Tricuspid Regurgitation

### 3.1. Determinants of Right-Ventricular Function

Normal RV performance depends on a complex interplay between systemic venous return (RV preload), pulmonary arterial load (RV afterload), pericardial constraint, intrinsic contractile properties of the RV free wall and IV septum, and ventricular interdependence [16]. Compared with the left ventricle (LV), the RV operates within a low-pressure, highly compliant pulmonary circulation, allowing it to maintain forward flow with substantially lower energy expenditure [17]. However, this physiological adaptation renders the RV particularly vulnerable to increases in afterload, which may rapidly lead to chamber dilation and reduced forward flow. This feature is central to the development of right HF in patients with TR, especially when pulmonary hypertension (PH) or left-sided heart disease coexist [18].

**Figure 1:**
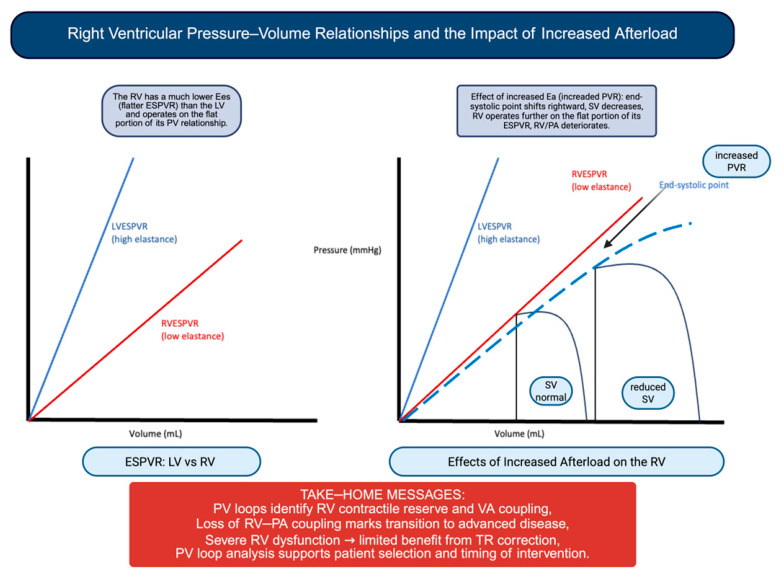
Schematic representation of left and RV PV loop relationships. The RV is characterized by a flatter end-systolic PV relationship compared with the left ventricle, reflecting lower contractile elastance. Under conditions of increased pulmonary vascular resistance, Ea increases, shifting the end-systolic point rightward, leading to increased end-systolic volume and reduced stroke volume. Abbreviations: Ea: arterial elastance; ESPVR: end-systolic pressure–volume relationship; LVESPVR: left-ventricular end-systolic pressure–volume relationship; PVR: pulmonary vascular resistance; RV: right ventricle; RVESPVR: right-ventricular end-systolic pressure–volume relationship; RV/PA: right ventricle/pulmonary artery; SV: stroke volume.

As illustrated in Figure 1, PV loop analysis provides a comprehensive representation of RV function and VA coupling. From a clinical perspective, it allows for the identification of patients with reduced contractile reserve, in whom correction of TR may not translate into clinical benefit.

### 3.2. Determinants of Right-Ventricular Afterload and Effects of Left-Sided Heart Disease on Pulmonary Circulation

Pulmonary vascular load is a key determinant of RV performance [19]. RV contractility is closely linked to pulmonary artery systolic pressure (PASP), underscoring the strong dependence of RV function on afterload conditions [19]. Compared with the LV, the RV exhibits a flatter end-systolic PV relationship, meaning that even modest increases in pressure may result in significant RV dilation and reduced stroke volume (SV) [19,20].

In the presence of left-sided heart disease, elevated left-atrial pressure further alters pulmonary vascular mechanics [21]. Increased left-sided filling pressures reduce pulmonary arterial compliance and increase the effective RV afterload beyond what is predicted by pulmonary vascular resistance (PVR) alone [22]. Thus, left-sided heart disease contributes to compromised RV function through combined effects on vascular resistance, compliance, and pulmonary vascular remodeling, often accelerating the progression toward right HF in patients with TR [22,23,24].

### 3.3. Ventriculo-Arterial Coupling

The interaction between RV contractility and vascular load can be described through the framework of ventriculo-arterial (VA) coupling [25]. This relationship is defined by the ratio between end-systolic elastance (Ees), reflecting ventricular contractility, and arterial elastance (Ea), representing vascular load [26] (Figure 1).

When RV contractility is no longer sufficient to match afterload, VA uncoupling occurs, leading to reduced mechanical efficiency and impaired forward flow [26]. In this context, invasive hemodynamic assessment may provide additional insights beyond non-invasive parameters. Reduced Ees/Ea values reflect impaired RV–PA coupling and have been associated with advanced disease stages and worse outcomes [26].

Moreover, elevated right-atrial pressure represents a hallmark of advanced right HF, reflecting impaired RV filling, while prominent v-waves may indicate severe TR and reduced right-atrial compliance. Quantitative indices such as the RV stroke work index further contribute to the assessment of RV performance, providing a direct measure of the ability of the RV to generate effective forward flow [26].

### 3.4. Coronary Perfusion of the Right Ventricle

In advanced stages, RV pressure overload may also impair coronary perfusion, further contributing to myocardial ischemia and progressive deterioration of the RV function [27].

**Figure 2:**
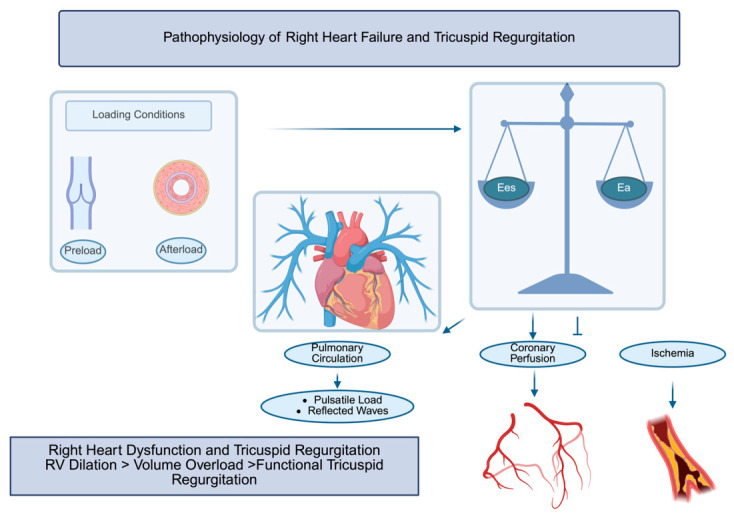
Pathophysiology of right heart failure and tricuspid regurgitation. Schematic representation of the key determinants of right-ventricular (RV) dysfunction and their interaction with TR. Abbreviations: Ea: arterial elastance; Ees: end-systolic elastance; RV: right ventricle.

Taken together, these mechanisms highlight the tight interplay of RV loading conditions, pulmonary vascular disease, and myocardial performance. In the setting of TR, progressive RV dilation and volume overload promote a vicious cycle of worsening regurgitation, declining RV function, and increasing systemic venous congestion.Importantly, once advanced RV dysfunction and end-organ damage are established, correction of valvular incompetence may no longer translate into meaningful clinical benefit. Therefore, understanding the transition from compensated TR to overt right HF is essential to defining the optimal timing of intervention and to identifying patients most likely to benefit from surgical or transcatheter therapies (Figure 2).

### 3.5. Practical Take-Home Messages for Clinical Decision Making

In this context, invasive pressure–volume (PV) loop analysis represents a valuable tool in clinical practice, providing a comprehensive assessment of RV performance and VA coupling beyond conventional parameters. From a physiological perspective, the RV is primarily a volume-adapted chamber, unlike the left ventricle, which is designed to sustain pressure loads. As a result, the RV is able to accommodate increases in preload with relative preservation of function, whereas it is poorly tolerant to increases in afterload, which may rapidly lead to dilation and reduced forward SV.

Within this framework, severe TR should be interpreted not only as a valvular lesion but also as a dynamic component of RV pathophysiology, acting both as a consequence and, in some cases, a modulator of RV dysfunction. In advanced stages, TR may transiently reduce RV pressure overload by providing a low-resistance outlet, although at the expense of effective forward flow. Therefore, the clinical impact of TR is highly dependent on the stage of disease, and therapeutic decisions should be guided less by regurgitation severity alone and more by the evaluation of RV function, contractile reserve, and pulmonary vascular load.

In this setting, PV loop analysis has a dual clinical role: it allows for the identification of patients with impaired RV contractile reserve who are unlikely to benefit from TR correction and enables a detailed characterization of biventricular remodeling after intervention. Consistently, recent data have shown that TR reduction after TTVI induces acute RV volume unloading with a compensatory increase in contractility and afterload, preserving forward SV and improving left-ventricular preload [23].

**Figure 3:**
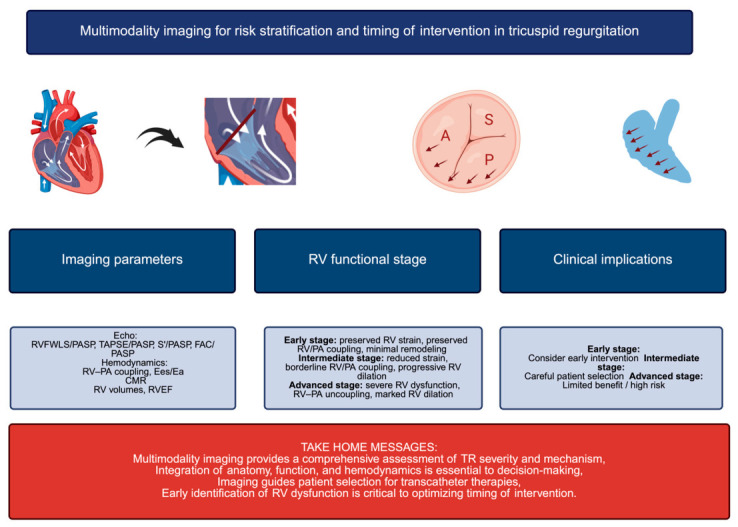
Multimodality imaging for risk stratification and timing of intervention in TR. Abbreviations: CMR: cardiac magnetic resonance; Ees/Ea: end-systolic elastance/arterial elastance; FAC: fractional area change; PASP: pulmonary artery systolic pressure; RV: right ventricle; RVEF: right-ventricular ejection fraction; RVFWLS: right-ventricular free wall longitudinal strain; RV–PA: right ventricle–pulmonary artery; TAPSE: tricuspid annular plane systolic excursion.

Modern management of TR increasingly relies on a multimodality imaging approach integrating transthoracic echocardiography (TTE), hemodynamic, and advanced imaging parameters. Beyond conventional measures, indices such as RV strain, RV–PA coupling, and the tricuspid annular plane systolic excursion (TAPSE)/PASP ratio provide important insights into RV function and its adaptation to afterload. In addition, cardiac magnetic resonance (CMR) represents the reference standard for the assessment of RV volumes and ejection fraction, allowing for a more accurate characterization of disease severity. These parameters may help refine risk stratification, identify early RV maladaptation, and support the timing of intervention, particularly in patients with borderline clinical profiles. As summarized in Figure 3, multimodality imaging enables an integrated assessment of TR, combining anatomical, functional, and hemodynamic information.

## 4. Acute and Chronic Right Heart Failure

### 4.1. Pathophysiology of Acute Right Heart Failure

Acute right HF develops when the RV is suddenly exposed to a marked increase in afterload or to an abrupt reduction in contractility [28]. Acute elevations in RV afterload may occur in conditions such as pulmonary embolism, severe hypoxemia, or acidemia, all of which increase PVR [28]. Alternatively, direct myocardial injury—such as RV ischemia, myocarditis, or postcardiotomy shock—may impair RV systolic function [28]. In both scenarios, RV SV may decline rapidly, leading to ventricular dilation and the subsequent worsening of TR. Progressive RV enlargement further exacerbates TR, creating hemodynamic deterioration [28]. RV dilation also impacts LV performance through ventricular interdependence [29]. As previously described, this mechanism reflects the mechanical interaction between the ventricles mediated by the interventricular septum and the pericardium. RV enlargement may cause septal flattening and leftward shift, impairing LV diastolic filling and contributing to systemic hypoperfusion [29].

Both diastolic and systolic interactions contribute to this process. Diastolic interaction arises from competition for filling within the constrained pericardial space, whereas systolic interaction reflects the contribution of LV contraction to RV systolic pressure generation, estimated to account for approximately 20–40% of RV systolic pressure [29]. Elevated right-sided filling pressures also impair myocardial perfusion. Increased venous pressure leads to coronary sinus congestion, reducing coronary blood flow and predisposing the RV to ischemia [29]. Moreover, systemic venous congestion adversely affects hepatic and renal function, promoting fluid retention and further worsening right HF [29].

### 4.2. Pathophysiology of Chronic Right Heart Failure

Chronic right HF develops gradually as a consequence of sustained pressure or volume overload [30]. The most common mechanism is progressive elevation in RV afterload due to PH, frequently secondary to left-sided heart disease. Chronic volume overload from right-sided valvular lesions, particularly severe TR, also contributes to disease progression [30].

Persistent hemodynamic stress initially induces adaptive RV remodeling characterized by cardiomyocyte hypertrophy and interstitial fibrosis, analogous to changes observed in left-sided HF [30]. During this compensated phase, the RV may maintain function despite increased loading conditions. However, with ongoing stress, the RV eventually transitions to a decompensated state, characterized by progressive myocyte loss, fibrosis, and chamber dilation [30]. Hemodynamically, this stage is associated with rising right-atrial pressure and worsening pulmonary vascular load. As cardiac output declines, PASP may paradoxically decrease despite persistently elevated PVR, reflecting advanced RV failure [29]. With an intact pericardium, progressive RV dilation leads to compression of the LV cavity, limiting LV filling and promoting equalization of biventricular diastolic pressures [29]. As a result, impaired LV filling is primarily driven by ventricular interdependence and pericardial constraint rather than reduced RV forward output alone [29]. Ultimately, the combined effects of RV systolic dysfunction, impaired LV filling, and systemic venous congestion lead to reduced cardiac output, diminished coronary perfusion, and progressive end-organ dysfunction [31].

## 5. Tricuspid Regurgitation in the Context of Right HF

**Figure 4:**
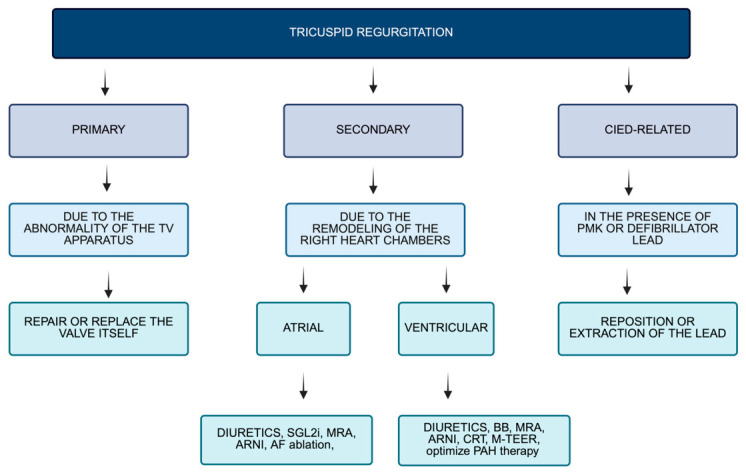
Classification of TR according to mechanisms and proposed practical approach to valve diseases considering pathogenesis. Abbreviations: AF: atrial fibrillation; ARNI: Angiotensin Receptor–Neprilysin Inhibitor; CIED: cardiac implantable electronic device; CRT: cardiac resynchronization therapy; MRA: Mineralocorticoid Receptor Antagonist; M-TEER: mitral trans edge-to-edge repair; PAH: pulmonary arterial hypertension; SGLT2: sodium–glucose transporter-2; TV: tricuspid valve.

TR is a heterogeneous condition encompassing distinct pathophysiological mechanisms that extend beyond simple valvular incompetence. The classification recently proposed by the PCR Tricuspid Focus Group and the Tricuspid Valve Academic Research Consortium (TVARC) categorizes TR into four main etiological groups: primary TR, atrial secondary TR (atrial-STR), ventricular secondary TR (ventricular-STR), and cardiac implantable electronic device (CIED)-related TR [32] (Figure 4). This framework relies predominantly on imaging findings to establish the diagnosis. Primary TR results from intrinsic structural abnormalities of the TV leaflets and accounts for the minority of cases (about 5%) [7]. In contrast, secondary TR occurs in the presence of anatomically normal leaflets and is driven by geometric and functional alterations in the right heart [9]. In particular, atrial-STR is mainly related to right-atrial enlargement and tricuspid annular dilation, whereas ventricular-STR, which is the more common form, is associated with right-ventricular dilation and dysfunction [33]. This distinction is clinically relevant, as atrial-STR has been associated with a more favorable prognosis compared with ventricular forms [9].

Although heterogeneous definitions of atrial-STR have been reported in the literature, recent consensus documents from TVARC and the PCR Tricuspid Focus Group have proposed standardized criteria to distinguish atrial and ventricular mechanisms [9,33]. More recent studies have further simplified this approach, emphasizing the role of the ratio between right-atrial and right-ventricular size (assessed by volumes or areas), integrated with left-ventricular and/or RV function, to refine classification and improve prognostic stratification [34]. CIED-related TR interests 10% of cases and represents a distinct entity in which the device lead contributes directly to regurgitation [35]. This may occur through mechanical interference with leaflet motion, entanglement within the subvalvular apparatus, or structural damage to the valve, or indirectly through pacing-induced RV dysfunction [36]. In this setting, three-dimensional imaging is particularly valuable for identifying the mechanism of valve–lead interaction [37]. Importantly, the clinical outcomes of TR differ substantially according to its underlying etiology [9].

## 6. Treatment of Right Heart Failure and Tricuspid Regurgitation

Relief of congestion represents a primary therapeutic objective in patients with right HF and more than severe TR [38]. Currently, no disease-modifying pharmacological therapies are specifically indicated for TR [38]. Nevertheless, in many patients, TR severity may be indirectly reduced through optimal management of associated conditions [39,40]. Notably, TR has been identified as a strong independent predictor of persistent congestion in patients hospitalized for acute HF [41,42]. Although diuretics remain the cornerstone of treatment for right HF, they primarily provide symptomatic relief without modifying disease progression, and robust randomized evidence is lacking [43,44]. Loop diuretics are commonly used to control volume overload; however, their effectiveness is often limited, particularly in advanced disease [43]. Renal dysfunction, frequently observed in severe TR, impairs diuretic efficacy through reduced renal perfusion and altered tubular drug delivery [43]. In addition, TR contributes to renal venous congestion, increasing interstitial and tubular pressures, which reduce glomerular filtration and promote diuretic resistance [44]. These mechanisms are further exacerbated by neurohormonal activation and worsening hemodynamics, including reduced cardiac output and elevated central venous pressure, which enhance sodium and water reabsorption [45,46]. Additional factors may further compromise diuretic response. Intestinal congestion may impair the absorption of oral diuretics; therefore, agents with higher bioavailability, such as torsemide or bumetanide, may be preferred [47]. However, the TRANSFORM trial did not demonstrate superiority of torsemide over furosemide in chronic HF, although patients were not specifically stratified according to TR severity [48]. In cases of inadequate response to high-dose oral therapy, intravenous administration of loop diuretics, either as bolus or continuous infusion, may improve hemodynamic by reducing PASP and increasing venous capacitance, possibly through prostaglandin-mediated effects [48,49,50]. Escalation of loop diuretic doses may be necessary to overcome resistance, although this approach is often less effective in patients with advanced chronic kidney disease. Combination strategies, including thiazide or thiazide-like diuretics (e.g., metolazone, hydrochlorothiazide, or intravenous chlorothiazide), allow sequential nephron blockade and may enhance diuretic response. More recently, the addition of acetazolamide or sodium–glucose cotransporter-2 (SGLT2) inhibitors has shown potential in improving decongestion [48,49,50]. In complex cases, aggressive diuretic strategies should be supported by a multidisciplinary approach, including pharmacological expertise, to optimize clinical outcomes.

### 6.1. Clinical Management of Tricuspid Regurgitation

The therapeutic approach to secondary TR is primarily directed toward the underlying disease [51]. Optimal management of comorbidities, including hypertension, diabetes, and chronic kidney disease, is essential, as well as correction of left-sided valvular disease and appropriate use of device therapies such as cardiac resynchronization therapy (CRT) when indicated [51].

In patients with HFrEF, guideline-directed medical therapy (GDMT) includes BBs, MRAs, Angiotensin Receptor–Neprilysin Inhibitors (ARNIs) (or ACE inhibitors/angiotensin receptor blockers), and SGLT2 inhibitors. In those with mildly reduced or preserved EF, SGLT2 inhibitors are recommended, with additional consideration of Mineralcorticoid Receptor antagonists, ARNIs, and incretin-based therapies in selected patients [43,51]. Although GDMT has not been specifically studied in severe TR, its beneficial effects on LV function, filling pressures, and pulmonary hemodynamics may indirectly improve RV function and reduce TR severity; however, direct effects on RV function remain uncertain [43].

Nonpharmacological interventions targeting left-sided heart disease may also improve TR. CRT has been associated with reverse RV remodeling and reduction in TR severity [52], and similar improvements have been observed after TAVI and M-TEER, although residual severe TR remains associated with worse outcomes [52,53].

Pulmonary vasodilator therapy is indicated in selected patients with confirmed pre-capillary PH after right heart catheterization, typically including phosphodiesterase-5 inhibitors, endothelin receptor antagonists, and prostacyclin analogues [54,55]. In acute RV failure, intravenous agents such as sildenafil or epoprostenol may reduce RV afterload. However, severe non-reversible pre-capillary PH remains a contraindication to tricuspid valve intervention [16,54,55,56].

In patients with AF, rhythm control strategies may promote reverse remodeling and reduce TR severity, although efficacy depends on disease stage [57,58]. CIED-related TR represents a complex scenario, as device leads may cause or worsen TR; lead extraction may improve or deteriorate valve function and requires careful multidisciplinary evaluation due to procedural risks [59,60,61,62,63,64,65].

### 6.2. Surgical Treatment of Tricuspid Regurgitation

Contemporary data have demonstrated a progressive improvement in surgical outcomes over time, including reductions in operative mortality, stroke, renal failure, reoperation, overall morbidity, prolonged ventilation, and length of hospital stay [66].

These are likely attributable, at least in part, to better patient selection and more accurate preoperative assessment of PH and RV function. In this context, prehabilitation strategies—consisting of inpatient medical optimization with aggressive diuresis and, when appropriate, inotropic support—have been associated with improved postoperative results [67]. Several preoperative factors have been identified as predictors of increased surgical mortality, including advanced New York Heart Association (NYHA) functional class (III–IV), nonelective surgery, and impaired liver dysfunction [68].

To date, no prospective randomized trials have compared ITVS with GDMT, and a clear survival benefit of surgical intervention has not been definitively demonstrated [69,70].

### 6.3. Transcatheter Tricuspid Valve Intervention

TTVI have emerged as a promising and rapidly evolving therapeutic option for patients with severe TR who are at high or prohibitive surgical risk [41]. Currently, four principal categories of TTVI are recognized: leaflet coaptation devices, annuloplasty systems, orthotopic tricuspid transcatheter valve replacement (TTVR), and heterotopic caval valve implantation (CAVI) [41,71,72,73,74,75,76]. Additional devices, such as spacers, may be considered within coaptation-based strategies, as they facilitate leaflet apposition by occupying the regurgitant orifice and reducing the effective regurgitant area [41]. More recently, transcatheter systems such as TriClip (T-TEER, Abbott) and EVOQUE (TTVR, Edwards Lifesciences) have received regulatory approval for use in patients with symptomatic severe TR despite GDMT, who are deemed appropriate candidates, with the aim of improving clinical outcomes [15,71,72,73].

To date, the comparative effectiveness of ITVS versus TTVI has not been directly evaluated, and no head-to-head comparisons between different transcatheter approaches—such as T-TEER and TTVR—are available. Instead, most studies have compared TTVI with GDMT. In this context, randomized trials such as the TRILUMINATE study (T-TEER vs. GDMT), the Tri-Fr (T-TEER vs. GDMT) and the TRISCEND II trial (TTVR vs. GDMT) have provided pivotal evidence supporting the use of transcatheter therapies [9,12,15,16,17,18,19,20,21,22,23,24,25,26,27,28,29,30,31,32,33,34,35,36,37,38,39,40,41,42,43,44,45,46,47,48,49,50,51,52,53,54,55,56,57,58,59,60,61,62,63,64,65,66,67,68,69,70,71,72,77] (Table 1). In parallel, the CLASP TR study evaluating the PASCAL system has further confirmed the feasibility, safety, and effectiveness of TEER, showing consistent reductions in TR severity and improvements in functional status in high-risk populations [78]. Overall, these studies have demonstrated that TTVI is associated with high procedural success and significant improvements in symptoms and quality of life, largely driven by effective reduction in TR severity. The stability of the reduction in symptoms has been demonstrated also in three-year follow-ups [74]. More in detail, T-TEER has been associated with excellent safety and consistent symptomatic benefit, whereas TTVR has shown the ability to achieve more complete elimination of TR, albeit at the expense of higher procedural complexity and increased rates of complications, including bleeding and need for permanent pacemaker implantation [71,72].

Eligibility criteria in major trials, including the TRILUMINATE study and the TRISCEND II trial, have required the presence of severe or greater TR confirmed by an independent TTE core laboratory [15,71,72]. In these studies, patients were typically considered at high surgical risk, as determined by multidisciplinary team assessment or elevated predicted surgical mortality scores [72].

Beyond randomized evidence, real-world data from large multicenter registries such as the TriValve registry have provided important insights into the performance of TTVI in routine clinical practice [73]. These data confirm the feasibility and safety of transcatheter approaches across heterogeneous populations, demonstrating consistent improvements in symptoms and functional status. At the same time, registry findings underscore that outcomes are strongly influenced by disease stage, particularly baseline RV impairment and the severity of venous congestion, reinforcing the importance of early referral and careful patient selection [73]. In this context, further evidence is awaited from ongoing randomized studies such as TRIC-I-HF-DZHK24 trial, which has specifically evaluated TTVI in patients with advanced right HF, a population traditionally considered at high risk and with potentially limited reversibility of disease [76].

In this context, appropriate patient selection and procedural planning represent critical determinants of procedural success and clinical benefit [51]. In particular, the choice between T-TEER and TTVR is largely driven by anatomical suitability and disease stage. T-TEER is generally preferred in patients with more favorable leaflet anatomy, a bileaflet or trileaflet valve morphology, a relatively small septolateral coaptation gap, confined prolapse or flail, low tethering height, and adequate leaflet tissue for grasping, where effective leaflet approximation can be achieved [51]. Conversely, TTVR may be more appropriate in the presence of complex valve morphology, including large coaptation defects, dense chordae or advanced leaflet tethering, where repair strategies are less likely to provide meaningful reduction in TR [51].

Anatomical constraints such as coaptation gap and leaflet tethering play a central role in determining procedural feasibility and device selection, while RV function and pulmonary hemodynamics further influence the physiological tolerance to intervention [51]. In particular, in patients with advanced RV failure, complete elimination of regurgitation—more frequently achieved with replacement strategies—may result in an abrupt increase in effective afterload and potential hemodynamic deterioration, the so called “afterload mismatch” [51]. Accordingly, the procedural strategy should be individualized, integrating anatomical characteristics, RV performance, and overall disease stage to balance procedural efficacy with physiological tolerance and optimize clinical outcomes.

## 7. Risk Stratification and Clinical Risk Scores in Tricuspid Regurgitation

**Figure 5:**
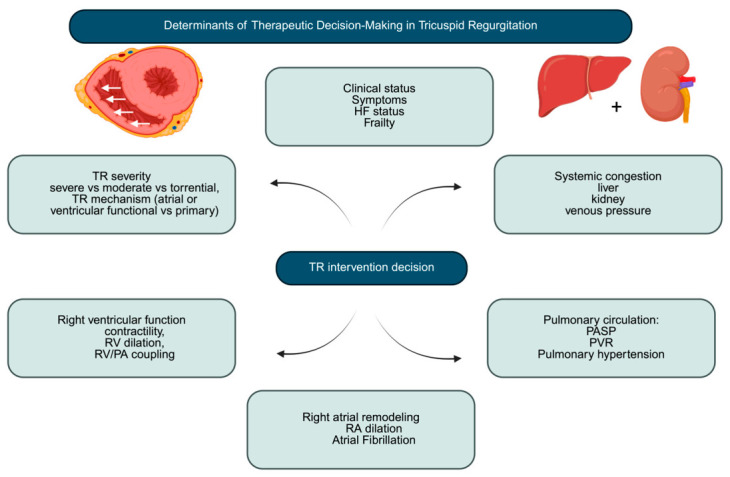
Determinants of therapeutic decision making in tricuspid regurgitation. Abbreviations: HF: heart failure; PASP: systolic pulmonary artery pressure; RA: right atrium; RV: right ventricle; PV/PA: right ventricle/pulmonary artery; PVR: pulmonary vascular resistance; TR: tricuspid regurgitation.

The need for dedicated risk stratification tools has become increasingly evident to better inform clinical decision making in patients with TR. Conventional surgical risk models are widely used but have shown limited accuracy in the more-than-severe-TR population, as they do not adequately account for key determinants of right HF [79,80]. To address these limitations, more recent efforts have focused on the development of disease-specific risk models that incorporate clinical, TTE, and biochemical variables closely related to the pathophysiology of the valvular disease. Beyond estimating perioperative risk, these tools aim to provide a more comprehensive characterization of disease stage, thereby facilitating identification of the optimal time for intervention (Figure 5).

### 7.1. STS-Score and EuroSCORE II

Risk stratification in ITVS has traditionally relied on general cardiac surgery scores, such as EuroSCORE II or the Society of Thoracic Surgeons (STS) score [79]. Although widely used, these models were derived predominantly from left-sided surgical populations and do not adequately describe the unique hemodynamic and systemic features of advanced TR [15]. Interestingly, the EuroSCORE II population comprised only 85 patients who underwent ITVS [79]. They failed to incorporate markers of right HF, which are central determinants of prognosis in this population [18]. Consequently, as shown in the study by Dreyfus and colleagues, both the logistic EuroSCORE and EuroSCORE II demonstrated limited predictive performance for in-hospital mortality after ITVS (C-index values of 0.67 and 0.63, respectively) [80].

### 7.2. LaPar Score

In an effort to improve the assessment of surgical risk in patients undergoing ITVS, LaPar et al. developed a dedicated risk score derived from a subset of the STS database including 2050 patients from the states of Virginia and Michigan for a total of 50 hospitals [81]. Although the authors should be commended for this important contribution, which represented a meaningful step toward improved risk stratification in patients referred for ITVS, the model presented important limitations as recognized by the authors themselves [81]. First, the study was based on an observational registry analysis; therefore the reported associations between risk factors and outcomes could not establish causal relationships. In addition, the limited clinical granularity of the registries prevented the inclusion of several relevant prognostic variables, such as RV function, markers of hepatic dysfunction, the underlying etiology of TV disease or infective endocarditis, and procedural details including the impact of aortic cross-clamping [81]. As a result, key determinants of outcomes after TTVI—particularly RV dysfunction and liver impairment—could not be incorporated into the risk model. Furthermore, the dataset was restricted to two U.S. states (Virginia and Michigan), potentially limiting the generalizability of the findings, and the model predicted only short-term perioperative mortality without long-term outcome assessment [81]. Finally, the proposed score did not undergo internal or external validation, leaving its performance in independent populations uncertain [81]. Furthermore, as also acknowledged by Dreyfus and colleagues, the score showed limited calibration in high-risk patients, with a maximum predicted mortality rate of only 34%, which may underestimate the risk in the most advanced clinical scenarios [80].

### 7.3. MELD Score

The Model for End-Stage Liver Disease (MELD) score, originally developed to stratify patients awaiting liver transplantation, has also been investigated as a predictor of mortality after TTVI [82]. In a cohort of 168 patients undergoing TTVI, the MELD score was shown to be associated with postoperative mortality; however, only a small subset of patients (*n* = 22) underwent ITVS [82]. The MELD score is calculated using three variables reflecting hepatic and renal function—international normalized ratio (INR), total bilirubin, and creatinine [82,83]. Nevertheless, its applicability in patients undergoing ITVS may be limited. In particular, a large proportion of these patients receive chronic oral anticoagulation therapy, including vitamin K antagonists or direct oral anticoagulants, which may artificially increase INR values and therefore reduce the interpretability and reliability of the score in this clinical setting [83].

### 7.4. TRIO Score and the Novel RV TRIO Score

More recently, the Tricuspid Regurgitation Impact on Outcomes (TRIO) score has been proposed as a clinical tool for risk stratification in patients with significant TR [84]. Specifically, it estimates 1- and 5-year mortality in the adult population [84]. This score was derived from a large retrospective cohort including 13,608 patients with moderate to severe TR identified at index TTE and followed for a median of 6.5 years [84]. Using multivariable Cox regression analysis, several clinical variables independently associated with all-cause mortality were identified, including age ≥70 years, male sex, creatinine >2 mg/dL, congestive HF, chronic lung disease, elevated aspartate aminotransferase levels, heart rate ≥90 beats/min, and severe TR. These variables were combined into a simple point-based score that demonstrated moderate discriminative ability for predicting mortality (C-statistic of 0.67) and allowed for stratification of patients into distinct risk categories. The performance of the model was confirmed in an independent geographically distinct cohort of 7138 patients [84].

However, given that the TRIO score is primarily based on clinical variables, subsequent studies have investigated whether TTE markers of RV function could further refine risk stratification in this population. In a retrospective cohort of 417 patients with moderate or more TR, quantitative parameters of RV function, including right-ventricular free wall strain (RVFWS) and RVFWS indexed to right-ventricular systolic pressure (PASP), were evaluated in addition to the TRIO score [85]. During a median follow-up of approximately four years, both impaired RVFWS (<18.6%) and reduced RVFWS/RVSP (<0.43%/mmHg), a non-invasive surrogate of RV/PA coupling, were independently associated with increased mortality even after adjustment for the TRIO risk score [84]. Incorporation of RVFWS/RVSP into the clinical model led to the development of the TRIO-RV score, which improved risk discrimination and reclassified a substantial proportion of patients initially categorized as low or intermediate risk into higher-risk categories [85].

### 7.5. TRISCORE

The recognition of the limitations of conventional surgical risk scores has led to the development of disease-specific models, among which TRISCORE represents the first tool specifically derived from patients undergoing ITVS. By integrating clinical, biological, and TTE parameters reflecting RV impairment, renal and hepatic impairment, and symptomatic burden, TRISCORE was designed to provide a more accurate estimation of operative risk in this high-risk population [79].

Beyond its predictive performance, TRISCORE has relevant clinical implications, extending beyond perioperative risk estimation to reflect the overall severity of disease. The score may identify a continuum ranging from early RV remodeling to advanced stages characterized by end-organ involvement. In this context, higher TRISCORE values are associated with more advanced disease profiles, in which the potential benefit of intervention may be limited [75]. Importantly, data from large registries such as TRIGISTRY have shown that patients with higher TRISCORE values derive limited benefit or no benefit from intervention, whether surgical or transcatheter, supporting its role in identifying advanced stages of disease and informing clinical decision making [75]. This reflects the pathophysiological scenario in which, when RV function is already severely impaired, TR acts as a “pop-off” mechanism; its correction removes this low-resistance outlet, increases effective afterload, and may further deteriorate RV performance [31].

From a clinical perspective, TRISCORE may therefore be integrated into a multiparametric framework to support patient selection and guide the timing of intervention. Lower-risk profiles may help identify patients more likely to benefit from earlier treatment, whereas higher scores may indicate advanced right HF with reduced reversibility, highlighting the risk of futility. Accordingly, the integration of TRISCORE with imaging parameters, RV–PA coupling indices, and markers of venous overload may contribute to a more individualized and stage-oriented approach to the management of TR.

In this context, we performed a synthesis of the available evidence to evaluate the performance and clinical implications of TRISCORE in patients undergoing ITVS across different populations and clinical settings [79,86,87,88,89,90].

#### 7.5.1. Application of TRISCORE in Surgical Population

The original derivation study by Dreyfus et al. established TRISCORE as a reliable predictor of in-hospital mortality, showing good discrimination (AUC 0.81) and calibration. Its prognostic value extended beyond the early postoperative phase, with a C-index of 0.78 for 1-year mortality, suggesting that the score reflects not only perioperative risk but also elements of overall clinical severity [80] (Table 2).

Subsequent validation studies have generally confirmed these findings. External validation in Spanish cohorts demonstrated high discriminative ability (AUC: 0.87), supporting the robustness of the score [87]. Similarly, Sala et al. reported sustained predictive performance over long-term follow-up, with discrimination exceeding 0.80 and a significant association with mortality (HR: 1.47) [88]. These observations suggest that TRISCORE may capture broader aspects of disease burden. In redo ITVS populations, TRISCORE maintained good discrimination (AUC: 0.83) and identified a clear risk gradient, with mortality ranging from 0% in low-risk patients to 25% in high-risk groups [86]. These findings support its utility in surgical risk stratification, although they derive from observational data.

Observational evidence has further explored the potential clinical implications of TRISCORE. In the study by Gwak et al., surgical intervention was associated with improved survival compared with medical therapy, particularly in patients with lower prognostic risk [89]. These findings suggest a possible role of TRISCORE in supporting therapeutic decision making; however, this application remains unproven and requires prospective validation.

Validation in non-European populations supports the generalizability of the score while also highlighting important differences in patient characteristics. In an Asian cohort, TRISCORE demonstrated good predictive accuracy for both in-hospital (AUC: 0.84) and long-term mortality (C-index: 0.77) [90]. Compared with European cohorts, patients were older, were more frequently female, and had higher prevalence of atrial fibrillation and hypertension, suggesting differences in underlying disease mechanisms [90]. Functional TR was also more prevalent, while the rate of TTVR was lower, reflecting differences in clinical practice and patient selection. Despite these variations, TRISCORE values were broadly comparable, suggesting a similar distribution of disease severity across populations [90].

Clinical outcomes also differed between cohorts. While the original TRISCORE population reported in-hospital mortality rates of approximately 10%, the Asian cohort showed lower in-hospital mortality (3.5%) and improved long-term survival, likely reflecting differences in patient selection and treatment settings [90].

Taken together, these findings support the use of TRISCORE as a useful tool for risk stratification in patients undergoing ITVS. The consistent observation of a risk gradient across score categories suggests a relationship between disease severity and outcomes.

#### 7.5.2. Application of TRISCORE in TTVI Population

The application of TRISCORE has recently expanded beyond surgical populations to include patients undergoing TTVI. In this setting, however, its performance appears more heterogeneous and overall less consistent [91,92,93,94]. Although the score retains some prognostic value, its ability to accurately predict in-hospital and long-term mortality is more limited. For example, data from Omran et al. suggest that TRISCORE may overestimate mortality in TTVI populations, likely reflecting differences between surgical and transcatheter cohorts [91] (Figure 2) (Table 3).

Similarly, studies by Gröger et al. and Vogelhuber et al. indicate that the predictive value of TRISCORE is more pronounced in patients with advanced disease, where higher score values are associated with worse outcomes [92,93]. However, its performance in low- and intermediate-risk patients is less well defined, limiting its utility as a comprehensive risk stratification tool in this setting. In addition, different studies have proposed varying cut-offs for high-risk patients (≥8 to ≥9), highlighting the lack of standardization and the potential need for recalibration in TTVI populations [91,92,93,94]. Data from large registries, such as the TriValve registry, further suggest that in transcatheter settings, TRISCORE may function more as a marker of advanced disease rather than as a precise predictor of procedural risk [94]. Its ability to identify patients with limited potential benefit from intervention may be clinically relevant, although this hypothesis requires further validation.

Despite its strengths, several limitations of TRISCORE should be acknowledged. Most available data derive from retrospective observational studies, making the score susceptible to selection bias and unmeasured confounding. Furthermore, TRISCORE was developed in surgical cohorts, which limits its generalizability to broader populations, particularly those undergoing TTVI. Additional sources of variability include heterogeneity in endpoints, follow-up duration, and reporting methods across studies. Formal calibration analyses are inconsistently available, and prospective validation in randomized settings is lacking. Finally, although TRISCORE incorporates variables related to RV function, it does not fully capture the complexity of right HF, including dynamic changes in RV–PA coupling, advanced imaging parameters, and evolving hemodynamic profiles.

Overall, while TRISCORE represents a valuable and disease-specific tool for risk stratification in ITVS, its performance appears less robust in transcatheter populations, and its broader clinical applications—including its role in disease staging and the timing of intervention—remain to be fully established.

### 7.6. Comparison Between Risk Scores

Overall, available risk scores in TR should be viewed as complementary rather than interchangeable, as they capture different aspects of patient risk. Conventional surgical models such as EuroSCORE II primarily estimate perioperative mortality and remain useful for procedural risk stratification but lack specificity for right heart pathophysiology [79]. The MELD score provides indirect insight into congestive state through hepatic dysfunction, although it is influenced by non-cardiac factors and does not reflect cardiac mechanics [82]. Procedure-oriented models such as the TRIO score offer simplified risk stratification in transcatheter populations but are limited by modest validation and incomplete integration of systemic and hemodynamic parameters [84,85]. Similarly, the LaPar score, although derived from tricuspid populations, remains constrained by limited generalizability and a focus on surgical cohorts [81]. In contrast, TRISCORE represents the most comprehensive disease-specific tool, integrating impaired RV performance, end-organ involvement, and clinical status, thereby providing a stage-oriented assessment of disease severity [79]. In clinical practice, an integrated approach combining conventional surgical scores for procedural risk estimation with disease-specific tools such as TRISCORE to assess disease stage and reversibility may offer the most informative framework to guide patient selection, optimize the timing of intervention, and identify patients at risk of futility in advanced disease.

## 8. Clinical Take-Home Messages

TR should no longer be considered a benign or secondary condition, as increasing evidence demonstrates its independent association with adverse clinical outcomes, including HF hospitalization and mortality. The management of TR has evolved from a predominantly surgical approach toward a more nuanced, patient-centered strategy integrating multimodality imaging, hemodynamic assessment, and individualized risk stratification.

A key concept emerging from current evidence is the importance of the timing of intervention. Correction of TR at an advanced stage of right HF when significant RV decompensation and hepatic and renal involvement are already established may provide limited clinical benefit. Conversely, earlier intervention—before irreversible RV remodeling and end-organ damage occur—may improve outcomes in appropriately selected patients.

Patient selection remains central to therapeutic decision making. Risk stratification tools such as TRISCORE and the others may support this process, although their application beyond surgical populations requires further validation (Table 4).

**Figure 6:**
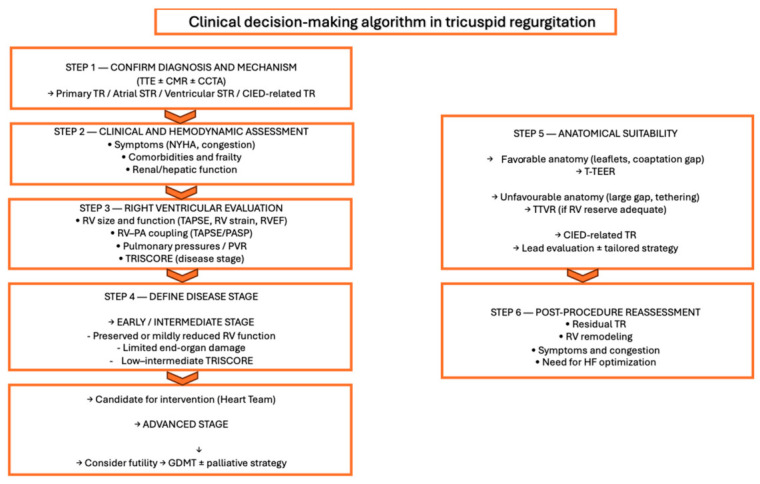
Clinical decision-making algorithm in tricuspid regurgitation. Abbreviations: CCT: cardiac computed tomography; CMR: cardiac magnetic resonance; HF: heart failure; RV: right ventricle; RVEF: right-ventricular ejection fraction; TR: tricuspid regurgitation; TTE: transthoracic echocardiography.

The rapid development of TTVI has expanded treatment options for patients at high surgical risk. T-TEER is associated with favorable safety and symptomatic improvement, whereas TTVR may achieve more complete elimination of regurgitation, albeit with higher procedural complexity. The choice between these strategies should be guided by anatomical characteristics, disease stage, and overall clinical profile. Finally, a multimodal approach integrating echocardiographic parameters (including RV strain and TAPSE/PASP), hemodynamic indices (RV–PA coupling), and advanced imaging such as CMR is essential to refining risk stratification and guiding the optimal timing of intervention (Figure 6).

## 9. Conclusions

TR represents a complex condition that is both a consequence and a driver of right HF. Overall, the integration of TRISCORE into clinical practice may support a more individualized, stage-oriented approach to the management of TR, helping to identify patients who may benefit from early intervention. Future studies should focus on prospective validation and on incorporating disease-specific risk stratification into clinical decision-making algorithms to optimize outcomes in this challenging population.

## Figures and Tables

**Table 1:** Key randomized and prospective studies on transcatheter treatment of tricuspid regurgitation.

Study	Design	Population	Intervention	Comparator	Primary Endpoint	Main Results	Ref.
TRILUMINATE Pivotal	Randomized controlled trial	Severe symptomatic TR, enrolled at 65 centers in the United States, Canada, and Europe	T-TEER (TriClip) + OMT	OMT	Hierarchical composite endpoint that included death from any cause or surgery; hospitalization and an improvement in quality of life as at the 1-year follow-up.	Significant improvement in QoL and TR reduction; no clear mortality benefit	[15]
Tri-Fr	Randomized controlled trial	Severe symptomatic TR in 24 centers in France and Belgium	T-TEER + OMT	OMT	Clinical composite endpoint at 1 year which includes change in functional class assessment, change in patient global assessment and a composite outcome of all-cause death, tricuspid valve surgery and time to hospitalization for HF.	No significant reduction in hard endpoints; improvement in symptoms and functional status	[12]
TRISCEND II (EVOQUE)	Randomized controlled trial	Severe TR	TTVR (EVOQUE)+OMT	OMT	Hierarchical composite endpoint (death, HF hospitalization, and improvement QoL).	Marked TR reduction and QoL improvement; higher procedural complexity	[71]
CLASP TR	Prospective single-arm study	Severe TR, high-risk patients	T-TEER (PASCAL system) + OMT	—	Safety and performance outcomes (TR reduction, NYHA, and QoL).	High procedural success; sustained TR reduction and symptom improvement	[78]

Abbreviations: HF: heart failure; NYHA: New York Heart Association; OMT: oral medical therapy; QoL: quality of life; TR: tricuspid regurgitation; T-TEER: tricuspid edge-to-edge repair.

**Table 2:** Performance and validation of TRISCORE in patients undergoing ITVS.

First Author	Year	Country	Study Design	No. of Patients	Procedure Type	Primary Outcome	Early Mortality (%)	1-Year Mortality (%)	C-Statistic (AUC)	Cut-Offs	Key Findings
Dreyfus et al., [80]	2022	12 French centers	Multicenter, retrospective cohort	466	ITVS	In-hospital mortality	10%	12%	0.81	≥5 high risk	TRISCORE shows excellent discrimination and calibration; also predicts 1-year mortality (C-index: 0.78)
Anguita-Gamez et al., [87]	2023	4 Spanish tertiary centers	Retrospective, observational study	252	ITVS	In-hospital mortality	10.3%		0.87 (95% CI 0.81–0.92)	≤4 vs. >4 (optimal cut-off); also risk gradient	TRISCORE identifies very-high-risk patients (mortality: 25% if >4 vs. 1.3% if ≤4)
Sala et al., [88]	2023	Registry data	Retrospective, single-center	176	ITVS	In-hospital mortality (and long-term mortality)	6.3%		0.82 (in-hospital); >0.80 up to 10 years	>5	TRISCORE shows strong performance in long-term prediction (HR: 1.47).
Gwak et al., [89]	2024	South Korea	Retrospective, single-center cohort	8874	ITVS vs. GDMT	5.2-year mortality				Surgical patients had a lower risk of death (HR: 0.38; 95% CI: 0.29 to 0.50) compared with medical management patients	ITVS was associated with higher survival rates in patients with moderate to severe TR and low prognostic risk
Kim et al. [90]	2024	2 Korean centers	Retrospective cohort	202	ITVS	All-cause mortality+ in-hospital mortality (concordance index, 0.77; cut-off value, 4)	3.5%	11.4%	0.77 (long-term mortality)/0.84 (in-hospital mortality)	4 (long-term)/3 (in- hospital)	TRISCORE strongly predicted both in-hospital and long-term mortality in Asian patients undergoing ITVS

Abbreviations: GDMT: guideline-directed medical therapy; ITVS: isolated tricuspid valve surgery; TR: tricuspid regurgitation.

**Table 3:** Performance and validation of TRISCORE in patients undergoing TTVI.

First Author	Year	Country	Study Design	No. of Patients	Procedure Type	Primary Outcome	Early Mortality (%)	1-Year Mortality (%)	Cut-Off	Key Findings
Omran et al., [91]	2022	2 high-volume centers (Heart Centers of Cologne and Bad Oeynhausen)	Double-center, retrospective	313	TTVR	In-hospital and 1-year mortality. The AUC for the ROC curve for 1-year mortality of TRISCORE was 0.750 and that for EuroSCORE II 0.553.	2.3%	20%	9	TRISCORE has good discrimination in patients undergoing TTVR, but it overestimates mortality.
Groger et al., [92]	2023	Ulm University Hospital	Single-center, prospective	180	TTVR	All-cause mortality (30-day and 1-year). TRISCORE was excellent (AUC for 30-day mortality: 90.3%; for one-year mortality: 93.1%) and superior to EuroSCORE II and STS-Score.	0–17.4%	0–52.2%	9	TRISCORE is a valuable tool to predict mortality after TTVR in the high-risk group.
Vogelhuber et al., [93]	2023	Germany	Retrospective, single-center study	302	TTVR	1-year composite (all-cause death + HF hospitalization).	NA	NA	NA	Higher TRISCORE is associated with worse outcomes (HR: up to 6.51 in high-risk group); procedural benefit attenuated in high-TRISCORE patients.
Adamo et al., [94]	2024	Trivalve registry	Retrospective, multicenter registry	634	TTVR ITVS	10-year all-cause mortality.	9.6%	NA	8	Surgery improves survival only in low-TRISCORE patients (HR: 0.27); repair beneficial in intermediate-risk patients and replacement harmful; no benefit in high-TRISCORE patients→ timing crucial.

Abbreviations: NA: tot available, TTVR: transcatheter tricuspid valve replacement.

**Table 4:** Summary of risk scores in TR: variables, strengths, and limitations.

Score	Derivation Population	Key Variables	Main Outcome Predicted	Validated Setting	Limitations	Clinical Application
EuroSCORE II [79]	General cardiac surgery	Age, renal function, LVEF, and comorbidities (16 variables)	Operative mortality	Broad cardiac surgery	Not specific for TR; underestimates risk in isolated TR	Generic risk score model used to predict mortality after cardiac surgery; it has no specific TV predictors.
LaPar Score [81]	Surgical TR cohorts	Clinical variables and comorbidities	Operative mortality	Surgical TR	Limited external validation	Limited to a single-center surgical cohort and focuses on perioperative mortality, not validated for TTVI.
MELD/MELD-XI [82]	Liver disease populations	Bilirubin, INR, and creatinine	Mortality (indirect)	TR with congestion	Reflects end-organ damage, not cardiac-specific	Developed to assess liver disease severity, it should be interpreted within a broader clinical context.
TRIO Score [84,85]	TR populations	Clinical + TTE parameters	Mortality	Mixed TR cohorts	Limited validation; heterogeneous populations	It does not fully capture disease severity.
TRISCORE [79,87,88,89,90,91,92,93,94]	ITVS patients	Age, NYHA, RV dysfunction, and renal/hepatic function	In-hospital and long-term mortality	Surgical TR	Derived from retrospective surgical cohorts; limited data in TTVI	TRISCORE represents the most comprehensive disease-specific model, although its application in contemporary transcatheter settings requires further validation.

Abbreviations: ITVS: isolated tricuspid valve surgery; LVEF: left-ventricular ejection fraction; NYHA: New York Heart Association; RV: right ventricle; TR: tricuspid regurgitation; TTE: transthoracic echocardiography.

## Data Availability

No new data were created or analyzed in this study.

## Abbreviations

The following abbreviations are used in this manuscript:

AF	atrial fibrillation
ARNI	angiotensin receptor–neprilysin inhibitor
a-STR	atrial secondary tricuspid regurgitation
BB	beta blockers
CAVI	heterotopic caval valve implantation
CCT	cardiac computed tomography
CMR	cardiac magnetic resonance
CIED	cardiac implantable electronic device
CRT	cardiac resynchronization therapy
Ea	arterial elastance
Ees	end-systolic elastance
GDMT	guideline-directed medical therapy
HF	heart failure
HFrEF	heart failure with reduced ejection fraction
ITVS	isolated tricuspid valve surgery
IV	interventricular
LV	left ventricle
MRA	mineralocorticoid receptor antagonist
M-TEER	mitral transcatheter edge-to-edge repair
PAP	pulmonary artery pressure
PASP	pulmonary artery systolic pressure
PH	pulmonary hypertension
PMs	papillary muscles
PV	pressure–volume
PVR	pulmonary vascular resistance
RV	right ventricular
RVFWLS	right-ventricular free wall longitudinal strain
SGLT2	sodium–glucose cotransporter-2
SV	stroke volume
TAPSE	tricuspid annular plane systolic excursion
TAVI	transcatheter aortic valve implantation
TR	tricuspid regurgitation
TTVI	transcatheter tricuspid valve intervention
T-TEER	transcatheter edge-to-edge repair
TTVR	transcatheter tricuspid valve replacement
TV	tricuspid valve
TVARC	Tricuspid Focus Group and the Tricuspid Valve Academic Research Consortium
v-STR	ventricular secondary regurgitation
